# Mapping social implications of wearables to monitor physical activity in palliative care – a qualitative approach

**DOI:** 10.1186/s12904-026-02213-x

**Published:** 2026-07-02

**Authors:** Natalie Öhl, Jordan Curry, Tobias Steigleder, Christoph Ostgathe, Maria Heckel, Cynthia C. Forbes

**Affiliations:** 1 Department of Palliative Medicine, University Hospital Erlangen, Friedrich- Alexander Universität Erlangen-Nürnberg (FAU), Werner-von-Siemens Str. 34, Erlangen, 91052 Germany; 2 Colorado State University, Physical Activity for Treatment and Prevention Lab, Fort Collins, Colorado US; 3 University of Technology Sydney, Sydney, New South Wales Australia; 4 Hull York Medical School, Wolfson Palliative Care Research Centre, University of Hull, Kingston upon Hull, UK

**Keywords:** Qualitative design, Activity, Wearables, Technology, Cancer, Oncology, Palliative care

## Abstract

**Background:**

Reduced physical activity is common among patients receiving palliative care. Wearables and other measurement devices offer opportunities to objectively monitor activity and support clinical decision-making and patient motivation. However, integrating such technologies into palliative care may affect care delivery, including routines, interactions and underlying principles. Given the strong emphasis on interpersonal relationships and patient-centeredness in this setting, wearables may entail specific social implications. This study therefore aims to map the social implications of using wearables to monitor physical activity in palliative care.

**Methods:**

Based on the CARE-HOUSE Framework, developed to conceptualise the social implications associated with digital health technologies, we investigated the potential social implications of using wearables (smartwatches and accelerometers) to monitor physical activity. Focus groups were conducted with palliative care researchers with clinical experience. After presenting background information on wearable activity monitors, participants were prompted to discuss prospective benefits, risks and unknown effects on stakeholders, interactions, social roles, and tasks in the context of healthcare practice. Data was analysed using content structuring analysis.

**Results:**

We conducted two focus groups with palliative care researchers with clinical experience (each *n* = 4; ~50 min duration) to map social implications of wearable technologies used to monitor physical activity in palliative care. The findings showed that wearable technologies introduce new tasks and organizational requirements in clinical practice. They may increase patients’ self-efficacy and motivation to remain active but can also provoke feelings of surveillance and influence interactions between patients, relatives, and healthcare professionals. Objective physical activity data may transform medical consultations by supporting assessment, and care planning. Furthermore, wearable technologies may support holistic palliative care by promoting physical activity, and therefore autonomy, and quality of life.

**Conclusions:**

Mapping the social implications of wearables to monitor activity can inform their implementation in palliative care. Addressing these implications is important to ensure that the devices support patient well-being. Future research should focus on translating these insights into practical guidance and incorporate the perspectives of both clinicians and patients.

**Supplementary Information:**

The online version contains supplementary material available at 10.1186/s12904-026-02213-x.

## Background

Physical activity is a key factor in maintaining and enhancing quality of life for patients receiving palliative care [1], particularly among those with advanced cancer [2, 3]. Palliative care, as defined by the World Health Organization (WHO) [4], includes patients with varying physical capacities and symptoms. In this paper, we focus on clinical palliative care, including palliative care units and hospital-based palliative care consultation services, often involving, but not limited to patients with advanced cancer.

The WHO defines physical activity as any bodily movement produced by skeletal muscles that requires energy expenditure and emphasises its importance for health and well-being [5]. Among palliative care patients, reduced physical activity, impaired functioning, and symptoms such as fatigue are common [6, 7]. Monitoring activity levels is crucial, as changes can indicate potential deterioration and guide timely interventions [8]. Functional status assessments are frequently used in clinical practice to evaluate treatment impact and symptom burden [9, 10].

Physical activity has well-documented benefits for palliative care patients. It can alleviate symptoms, reduce pain, promote relaxation and comfort, boost strength and energy levels, enhance well-being and reduce fatigue [6, 11]. It supports patients in performing activities of daily living, even as their abilities decline [7, 12–14], and can enhance self-esteem and determination, helping maintain or regain independence [15, 16]. Additionally, physical activity fosters motivation, security, hope, and relief, allowing patients to live with dignity [7, 17] and facilitating social participation [12, 15, 16].

Wearable technologies offer one approach to monitor and support physical activity. These compact, portable devices provide information to users and enable interactive engagement [18], often via body sensors that detect and record physiological processes [18]. Their unobtrusive design allows passive, long-term use [19], making them particularly suitable in palliative care. Wearables can measure physical activity parameters such as steps, active minutes, pulse oximetry, and heart rate, as well as variables such as sleep, posture, energy expenditure, or gait [19, 20]. Continuously monitoring patient-related parameters in real-time, even in real-world settings [21], provides clinically relevant longitudinal data that are objective and free from the errors and biases of self-reporting [19].

With technological advancements, wearable devices are increasingly applied across medical settings [18], including oncology [8, 19]. In palliative care, the use of wearable technologies has been studied for several purposes, for example, remotely monitoring patient health [21] or as a prognostic tool [22, 23]. This study focuses on two types of wearables: smartwatches, for example, Fitbit™, and accelerometers worn mid-thigh, for example, activPAL™. Both are commercially available and have been evaluated in several studies to measure physical activity in different populations in health care [24–26].

Data on physical activity can support decision-making and evaluate the suitability and effectiveness of therapy [19], allowing more proactive and personalised care [8]. At the same time, it might bring motivation and awareness for activity, but also improved self-awareness for patients [27]. Hence, wearables have the potential to enhance the care of palliative patients. However, implementing wearables can alter routines, workflows and interactions, leading to complex social effects [28, 29]. In palliative care, which emphasizes social inclusion and interpersonal connections, health technologies may have unique social implications [30, 31]. In this study, social implications refer to the effects of technologies on the human aspects of care, including stakeholders, their roles and interactions, as well as care processes. Excessive reliance on technology could risk dehumanization or compromise the essential human connection central to palliative care [32, 33]. Careful examination of these social implications is therefore essential to align technology use with core palliative care principles [30, 34].

The CARE-HOUSE Framework supports this endeavour by conceptualising the social implications of digital health technologies in palliative care [35, 36]. The framework highlights key dimensions of care practice that may be affected by digital health technologies, including care principles and objectives, involved stakeholders and their roles, interactions, tasks, processes, and contextual factors. By considering potential benefits, risks, and unintended effects across these dimensions, the framework supports a comprehensive analysis of social implications in palliative care settings.

The social implications of wearables to monitor physical activity in palliative care and their social effects on care practice have not been studied before. The aim of this study is to map social implications of wearables to monitor physical activity in palliative care clinical practice in order to support their responsible use.

## Methods

### Design

We used a qualitative design, informed by a constructivist epistemological approach [37], to map social implications of wearables in palliative care. We conducted two focus group discussions [38–40] with palliative care researchers with clinical experience in September 2023. The study received approval from the ethics committee on August 07, 2023; Hull York Medical School Research Ethics Committee, 22-23.66. The study process adhered to COREQ guidelines [41], ensuring transparency and rigor in reporting qualitative research.

### Setting and participants

This study included palliative care researchers with clinical experience from a Palliative Care Research Centre in the United Kingdom. Potential participants (*n* = 36) were selected and approached purposively by e-mail by CF. We included established palliative care researchers from relevant disciplines who have extensive publication records in their respective areas of expertise and clinical experience. Participants’ combined expertise in clinical processes and research made them well suited to discuss the social implications of wearable technologies in palliative care, as they were familiar with care practice while also understanding relevant conceptual and research considerations.

### Data collection

A focus group guide was developed and pilot tested based on the dimensions of the CARE-HOUSE Framework [35, 36]. The guide incorporated all dimensions identified by the CARE-HOUSE Framework as relevant for analysing social implications.

To structure the discussion and ensure that each dimension was addressed, the individual dimensions of the CARE-HOUSE Framework were presented sequentially on a poster during the focus groups. The following dimensions were introduced:

- Interactions.
- Social roles.
- Tasks.
- Stakeholders.
- Context conditions (societal, organizational, structural, professional).
- Principles and objectives of palliative care.

Within each dimension, participants were invited to reflect on potential benefits, unknown effects, and conflicts, risks, or adverse effects related to the use of wearable activity monitors in palliative care.

The focus groups took place on September 20, 2023, and September 27, 2023, at the Wolfson Palliative Care Research Centre – Hull York Medical School in Kingston upon Hull, United Kingdom, with a duration of 50 min each, and were audio-recorded. Focus groups were facilitated by NÖ, a research associate and health economist in the Department of Palliative Medicine at the University Hospital in Erlangen, Germany, in consultation with CF, researcher in exercise oncology and JC, research associate in exercise oncology both at the University of Hull, United Kingdom. The facilitators received training prior to conducting the study. Focus group participants knew each other well; NÖ was personally little-known to the participants; CF and JC were well known by the participants. We provided background information on the study, the collaboration of the two institutions and the relevance of social implications of health technologies. We presented basic technical and practical details on wearables to monitor physical activity, such as the features of application form, design, usability, and functions, and we showed such a device (activPAL™) for illustrative purposes.

### Data analysis

**Fig. 1:**
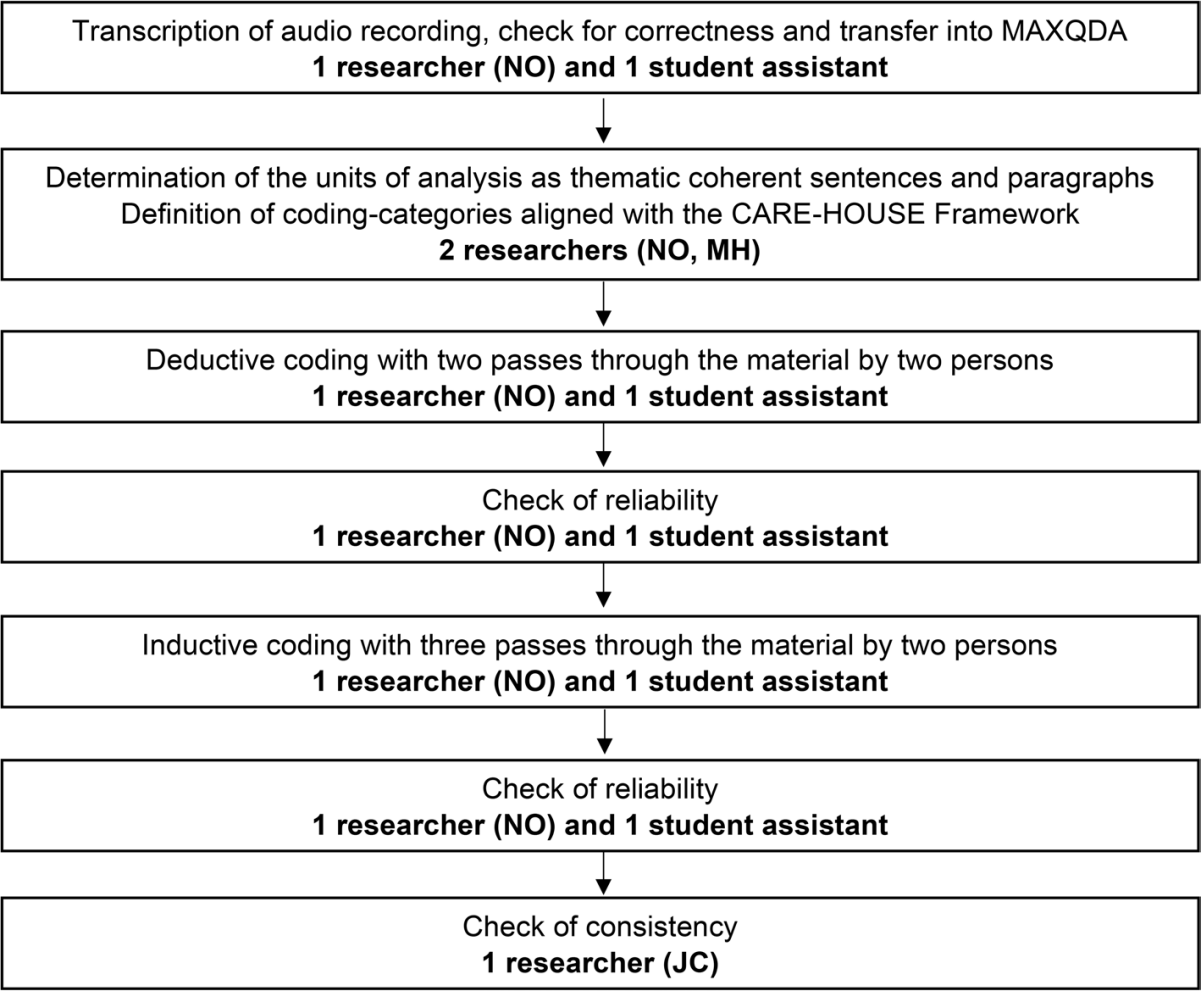
Data analysis using the method of structuring content analysis set out by Mayring [43, 44]

The audio was transcribed afterwards by a student assistant according to the transcription guide of Dresing and Pehl [42] and checked for correctness by NÖ. Transcripts were not returned to participants for comment and/or correction but were checked by a student assistant and NÖ for correctness. NÖ and a student assistant conducted structuring content analysis using content categories as defined by Mayring [43, 44] (Fig. 1). Structuring content analysis was chosen as the study aimed to systematically examine the data against the predefined theoretical dimensions of the CARE-HOUSE Framework, while still allowing for the identification of emerging subthemes within these categories [43, 44]. The analysis can be characterised as qualitative descriptive with a theory-guided orientation.

Coding took place using a subsumption strategy [43, 44] based on coding guidelines, assigning data to main categories and subcategories. We used the components of the focus group guide and the CARE-HOUSE Framework as deductive main categories and inductively added further main and subcategories. Two checks of reliability [43, 44] were conducted during the coding process to enhance intersubjectivity. The two coders compared their coding decisions, discussed discrepancies, and refined the coding system accordingly. In a final check of consistency [43, 44], a third researcher (JC) reviewed the coded material to ensure that categories were applied consistently across the dataset. This included verifying that similar text passages were coded identically, that no contradictory assignments occurred, and that the category system was internally coherent. The MAXQDA software (version 20) was used for the analysis of qualitative data [45].

The coding scheme (see supplements) included the following categories:

- Principles and objectives of palliative care (holistic treatment; prevent and relieve suffering; improve or maintain quality of life).
- Stakeholder level: patients (physical activity; life circumstances; burdens and challenges; needs and preferences), relatives (needs and preferences), health care professionals (skills and knowledge, burdens and challenges, needs and preferences), technology (design/location, purpose, quality, usage, functions).
- Delivery of care level: interactions (patient-physician interaction, patient-relatives interaction, professionals-relatives-patient interactions, human-technology interaction), social roles (conception of others, self-conception), tasks (general tasks in terms of movement, tasks in terms of technology),
- Context (societal, organizational, structural, professional).

## Results

### Sample characteristics

The focus groups had four participants each (*n* = 8), with no one dropping out, with the following characteristics (Table 1).

**Table 1:** Characteristics study population

		Focus group 1 *n* = 4	Focus group 2 *n* = 4	Total *N* = 8
**Gender**	male	2 (50%)	2 (50%)	4 (50%)
	female	2 (50%)	2 (50%)	4 (50%)
	diverse	0 (0%)	0 (0%)	0 (0%)
**Age**	under 20 years	0 (0%)	0 (0%)	0 (0%)
	20–30 years	3 (75%)	0 (0%)	3 (37,5%)
	31–40 years	0 (0%)	3 (75%)	3 (37,5%)
	41–50 years	0 (0%)	0 (0%)	0 (0%)
	51–60 years	1 (25%)	0 (0%)	1 (12,5%)
	over 60 years	0 (0%)	1 (25%)	1 (12,5%)
**Education**	Medicine	1 (25%)	1 (25%)	2 (25%)
	Medical Science	1 (25%)	1 (25%)	2 (25%)
	Exercise physiology	1 (25%)	0 (0%)	1 (12,5%)
	Sport science	1 (25%)	0 (0%)	1 (12,5%)
	Global health policy	0 (0%)	1 (25%)	1 (12,5%)
	Dietetics	0 (0%)	1 (25%)	1 (12,5%)

### Mapping social implications

**Fig. 2:**
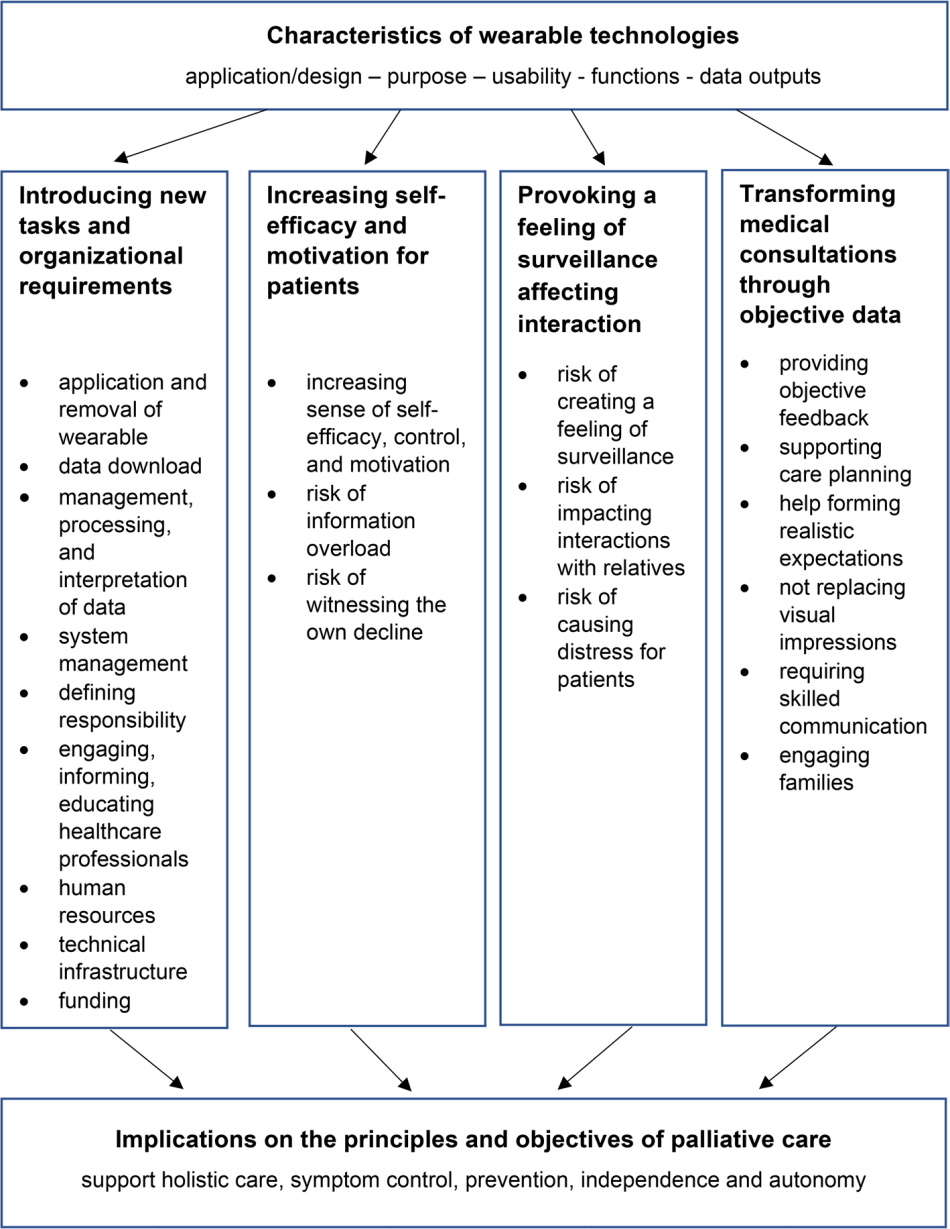
Mapped social implications of wearables to monitor physical activity in palliative care

Within the defined areas (interactions, roles, tasks, and context), we identified the following social implications of wearables for monitoring physical activity in palliative care (Fig. 2).

### Characteristics of wearable technologies

There are diverse types of wearable technologies to monitor physical activity, differing in application/design, purpose of use, usage, functions, and data output. In the focus groups, two types were discussed: *smart-watches*, (i.e., Fitbit™ or Apple Watch™), and *accelerometers* worn mid-thigh (i.e., activPAL™).

Regarding design and application, participants noted that *smart-watches*, worn on the wrist, may inaccurately register arm movements (e.g. gesturing while seated) as physical activity. Regular charging was seen as a potential source of data gaps, particularly relevant in palliative care patients who may experience cognitive burden and forget to put the device back on. Combining smartwatch data with short self-reports was considered a minimally intrusive way to support interpretation. In contrast, activPAL™ devices, worn continuously on the mid-thigh, were perceived as enabling more accurate and uninterrupted data collection. Minor discomfort (e.g., when lying on the device), was mentioned, but participants described it as discreet and likely to be forgotten after a short adjustment period.

Wearables also vary in purpose of use. Participants suggested that feedback on physical activity (e.g., sit-to-stand transitions) could help tailor interventions. *Smartwatches* offer real-time feedback and reminders, which were considered particularly useful for behavior modification, especially in outpatient settings with higher levels of functioning. In contrast, *accelerometers* such as activPAL™ might be suited for longer-term monitoring. Although patients may not always have direct access to real-time data, retrospective analyses (e.g., seven-day overviews) were seen as beneficial in both inpatient and outpatient care.

 Usability of different types of wearables varies and was identified as relevant for social implications, as it affects routines, practices, and interaction with the technology. *Accelerometers* require downloading data to a computer, which was considered labor- and time-intensive. For continuous use, activPAL™ must be covered with a waterproof sheath (Tegaderm) dressing, which may impair utility [46]. Given fragile skin in palliative care patients, participants expressed concerns about discomfort, skin irritation, allergic reactions, and practical difficulties (e.g., the dressing catching on clothing).

Finally, functions and data outputs differ. Participants noted that *smartwatch* algorithms are typically based on healthy populations and may not reflect the capacities of palliative patients, potentially leading to discouragement and underscoring the need for individualized metrics. *Accelerometers*, by contrast, provide more detailed movement analyses, including posture and body position (e.g., lying, sitting) and directional changes, allowing for a more nuanced understanding of activity pattern.

### Introducing new tasks and organizational requirements

Focus group participants stated that implementing wearables to monitor physical activity in palliative care would introduce new tasks and responsibilities for healthcare professionals, as well as organizational requirements (CARE-HOUSE Framework dimension: Tasks and organizational context). The implications depend on the setting; here we focus on clinical palliative care practice.

Participants identified application and removal of devices - particularly activPAL™ - as key practical challenges. Applying Tegaderm on activPAL™ may be technically demanding due to its thinness and tendency to wrinkle. Although experienced healthcare professionals are generally familiar with such procedures, additional competency assessments may be required, especially for healthcare assistants. Application and removal were considered relatively low in time burden, as Tegaderm™ is only replaced every seven days. However, removal may be challenging on hairy or fragile skin, requiring careful handling. The task also entails responsibility for regular skin checks to ensure safety. While perceived as a minor additional task, participants emphasized the need to clearly define who is responsible and whether trained non-professionals could perform it, particularly in outpatient settings. With basic training, participants did not consider this a problem. *“So*,* I think from the perspective*,* who applies it*,* once they are trained*,* which should be really basic training*,* I think that should not be any major burden.” (B3)*.

Some technologies, such as activPAL™, also require manual data download after the monitoring period, adding another task depending on the device used.

Participants viewed data management, processing, and interpretation as the most critical new responsibility. Key questions include who owns the data, who analyzes it, and for what purpose (e.g. monitoring, exercise prescription, assessing limitations). Given existing workload pressures, allocating responsibility was seen as challenging. Ideally, one professional group should take the lead in interpreting data and communicating relevant findings to others. Physicians, physiotherapists, and occupational therapists were identified as potential users, depending on whether the goal is baseline assessment, progress monitoring, or tailored intervention.

System management adds further responsibilities, including securing, importing, and managing data, as well as overseeing device identification.

Across all tasks, participants stressed the importance of clearly defining responsibility and accountability before implementation. Without structured role allocation, additional responsibilities may lead to dissatisfaction, conflict, or reduced engagement. Even small tasks may be perceived as burdensome in already strained work environments. Participants also expressed concern that staff might feel implicitly criticized if low patient activity levels are highlighted. *“We are like telling nurses that their patients are not moving enough*,* that we should get them to move more. I think that might be like ´Oh*,* we have enough to do already*,* we cannot spend twenty minutes walking with Mr. Smith on the corridor´.” (B1)*.

Successful implementation therefore requires organizational preparation. Participants emphasized the need for comprehensive explanation, education and training on device application, data download, and interpretation, as well as troubleshooting technical issues. Immediate support in case of malfunctions was considered essential to prevent patient anxiety.

Beyond training, fostering engagement and demonstrating benefit were viewed as crucial. Healthcare professionals are more likely to adopt wearables if they clearly understand their purpose and added value for patients. If benefits are unclear, resistance may arise, particularly in vulnerable patient populations. *“I guess you need to show that it is worth everything to collect that data and it is worthy of what is already collected anywhere that you probably got available”. (B2).*

Adequate human resources and staffing are seen as essential, as high workloads and staff shortages may hinder both physical activity promotion and technology adoption.

Implementation also requires robust technical infrastructure, including compatible software and dedicated support personnel. Introducing additional digital systems may create organizational barriers and lead to professional overload, especially where multiple systems already exist. *“So*,* there is already so many different systems that health care professionals are having to get information from*,* then to add another one into that could be an organizational barrier there”*. (B3). Ensuring firewall compatibility and secure data management with clear protocols was seen as essential.

Organizations need to secure funding not only for purchasing the devices but also for software, training and ongoing support.

Participants noted that wearable data could extend beyond single institutions, enabling cross-setting use and contributing to broader interoperability and e-health development.

### Increasing self-efficacy and motivation for patients

Focus group participants identified enhancing patients’ self-efficacy and motivation to remain active as a key advantage of wearables (CARE-HOUSE Framework dimension: involved stakeholders and their roles). In palliative care, rapid deconditioning is common, and patients may adopt a mindset of being sick and being “*in the role of a patient*,* they think they need to sit in their chair or lie on their bed*” (B4). This leads to feelings of dependence, with patients often believing their role is merely to wait rather than act. Wearables were seen as a potential means to counter this by fostering a sense of control and encouraging activity.

Because *smartwatches* are visible and provide real-time feedback, participants suggested that patients may frequently check their data, track their movements, and adjust their behavior accordingly. Engaging with activity data may strengthen motivation and increase physical activity. Patients may feel more in control of their health and experience a sense of self-efficacy, which could preserve autonomy/independence.

At the same time, participants expressed concerns about information overload, particularly among anxious or older patients. Continuous visibility of activity data may cause frustration if goals are not achieved and could increase pressure or anxiety. In contrast, *accelerometers* do not provide constant feedback, which may reduce stress and information overload. However, they may also have less impact on behavior change compared to *smartwatches*.

Participants further noted that repeatedly confronting patients with evidence of physical decline could be demoralizing. Observing deterioration through technological metrics might be perceived as “*hugely unhelpful*” (B1) and a potential “*trigger*” (B2), especially when efforts do not translate into measurable improvement, possibly leading to loss of hope or disengagement.

### Provoking a feeling of surveillance and affecting interactions

Participants expressed concern that monitoring physical activity through wearables could create feelings of surveillance (CARE-HOUSE Framework dimension: Interactions). Patients may feel uncomfortable being tracked, particularly in outpatient settings where resistance to perceived monitoring may be stronger. While awareness of monitoring might encourage activity, it could also evoke a “*big brother effect*“ (B1). It remained unclear whether such discomfort would persist or diminish as patients become accustomed to the device.

Depending on the wearable type, caregivers and support networks may access activity data, potentially impacting interactions. With real-time *smartwatch* data, relatives might closely monitor activity levels and interpret reduced movement as a need to push the patient further.

Participants emphasized that witnessing a loved one’s physical decline is often distressing and overwhelming. In their desire to help, relatives may focus strongly on encouraging activity when they see objective data. This may lead them to promote levels of activity that are no longer realistic, reflecting difficulty in accepting the patient’s current limitations. Family members often hold on to memories of the patient at their best and may struggle to adjust expectations accordingly. Such denial can result in pressure on the patient, causing stress, guilt, and frustration. When patients are unable to meet these expectations, they may experience feelings of failure and demotivation, negatively affecting their emotional well-being. Participants therefore expressed concern that, although *smartwatches* provide valuable insights, they may also complicate family dynamics and add emotional strain: *“Relatives would often pressure their loved ones and be like ´Oh*,* you are not moving enough*,* you are just not trying´. ”* (B4).

In contrast, *accelerometers* were perceived as offering greater privacy, as they do not display activity data directly to relatives and therefore less intrusive in social interactions. This may allow patients to keep their activity levels more personal if they prefer not to share this information.

Participants also noted that relatives do not always push for more activity. In some cases, they prioritize rest, sometimes influenced by cultural norms regarding illness. In such situations, wearable data could help demonstrate patients’ remaining capabilities and support a shared understanding of the benefits of appropriate activity.

Finally, participants stated that feelings of surveillance may also arise in the patient–professional relationship. Knowing that healthcare professionals monitor movement data could create a sense of surveillance, making some patients feel uncomfortable with the idea that their actions are being closely observed. *“Some people might not like the idea that someone is checking in on them and whether they are doing certain activities.” (B4)*.

### Transforming medical consultations through objective data

Focus group participants stated that objective physical activity data from wearables (both smartwatches and accelerometers) could enhance consultations and patient-physician interactions (CARE-HOUSE Framework dimension: Interactions). By providing measurable feedback, physicians could offer more targeted advice, such as tailored exercises, and identify areas for improvement. Objective data may also increase transparency, helping to reconcile discrepancies between patient’s perceived and actual activity levels - particularly when physicians are not yet familiar with the patient.

Participants highlighted the value of such data in care planning discussions. It could support evidence-based explanations of treatment options and help contextualize changes in activity levels, for example by demonstrating that decline may reflect disease progression rather than neglect. At the same time, participants cautioned against over-reliance on objective measures, noting that performance data can be interpreted subjectively and should not replace clinical judgment or flexibility in care.

Objective movement data may also support the development of realistic expectations regarding patients’ physical capabilities. Perceptions of activity levels often vary widely, and discrepancies may exist between patient and physician assessments [47, 48]. Wearable data can provide clarification and a reality check, although this may sometimes be difficult for patients to accept.

Participants emphasized that clinical observation remains indispensable. Directly observing a patient and getting a visual impression —for example, seeing them walk informally—provides insights that technology cannot fully replace. Wearables were therefore viewed as complementary, offering additional information between consultations rather than substituting professional assessment.

Skilled communication was considered essential when using wearable data. Results may be misunderstood or misleading without proper explanation, and sensitive handling is required to avoid discouragement or harm. As one participant noted, in a position of trust, such data are *“one tool in your toolbox”* (B2), but if misused, they may be harmful.

Wearables were also seen as a means to engage family members constructively in care. Relatives could be encouraged to support shared activities, such as accompanying the patient outdoors. However, participants stressed the importance of careful debriefing and clear communication to ensure that relatives understand the patient’s limitations and avoid placing undue pressure on them.

### Implications on the principles and objectives of palliative care

Focus group participants emphasized the critical role of physical activity in palliative care. Maintaining mobility and encouraging regular movement for as long as possible were described as key objectives in advanced disease. Physical activity was associated with benefits for mental wellbeing, social interaction, and prevention of complications. The use of wearables was primarily linked to the aim of promoting and monitoring physical activity. Participants connected these aspects to core principles of palliative care, particularly its holistic and personalized orientation. As one participant stated: *“It also hits the principles of palliative care because the benefits of activity are truly holistic and not just physical”. (B2).*

Participants emphasized that wearables could support symptom control and maintaining quality of life. *“If you can improve symptoms like (…) fatigue or breathlessness through activity it would be a highly valued objective for quality of life and functional status.”* (B3).

Focus group participants also highlighted, that wearables could support in preventive measures. As palliative care extends beyond end-of-life and aims to enhance quality of life throughout illness, early use of wearables could help patients remain active, engaged, and connected to meaningful activities. As one participant stated: “W*e want to support people living their best they can until their final days.”* (B1). This was seen as particularly relevant for patients with non-malignant conditions who experience long-term symptoms and may not yet access palliative care. For this group, a functional approach facilitated by wearables could be particularly beneficial, potentially slowing deterioration and improving functional status, where even modest improvements may have meaningful impact.

Enhanced activity may also strengthen independence and autonomy by fostering a sense of control, which is particularly important in palliative care. Even modest functional gains—such as being able to reach a bus stop in the outpatient setting—were described as meaningful improvements in daily life.

At the same time, in palliative care, improvements often occur within very small margins, and gains may be minimal. Wearables should therefore only be used when a clear benefit for the patient is evident, to avoid demotivation or undue pressure. *“If you are really fatigued and do not want to*,* you are going to build up kind of some level to stand against ´I don’t want to move*,* I want to just lay here cause I’m tired´. And that is okay*,* encouraging that when they want to.” (B1)*.

## Discussion

This study mapped the social implications of integrating wearable technologies to monitor physical activity into clinical palliative care practice. The findings highlight both potential social benefits and challenges that should be considered when implementing such technologies to ensure their use aligns with core principles of palliative care. The integration of wearable technologies in palliative care therefore represents not only a technical innovation but also a social intervention that may influence care practices and the care environment.

As wearable technologies are not yet widely integrated into routine palliative care, this study mapped their potential social implications prospectively. Demonstrating a wearable device (activPAL™) during the focus groups supported participants´ understanding of its practical application and facilitated discussion about its possible use in clinical settings. Identifying potential benefits and challenges at an early stage may help inform implementation strategies that are sensitive to the specific needs of palliative care [30].

One key social implication identified in this study relates to the introduction of new tasks and organizational requirements. These findings need to be considered within the broader context of technology implementation and the integration of wearable devices into existing clinical and organizational structures. Previous research has highlighted the importance of systematic and iterative implementation strategies when introducing digital health technologies into clinical workflows [19]. Several challenges have already been identified, including issues related to data management and interpretation, patient engagement, integration into existing technical infrastructures, as well as privacy and ethical concerns [8, 27]. Our findings support these observations and further underline that new tasks and organizational processes affect healthcare professionals and other stakeholders, and these impacts must be considered. In addition, the costs associated with implementing such technologies extend beyond their initial purchase, highlighting the need to evaluate their overall impact and added value for patient care [19].

Other social implications identified in this study relate to the role of patients and to interactions between patients, relatives and healthcare professionals. These implications highlight both potential benefits and risks, suggesting that wearable technologies may support key principles of palliative care [49, 50] —such as symptom management, prevention, autonomy, and independence—while also requiring careful and context-sensitive implementation.

In clinical practice, various interventions already aim to promote physical activity among palliative care patients, including physiotherapy programs [51], movement therapy programs [52], dance movement psychotherapy [53], and multimodal interventions combining exercise, nutrition, and palliative care [54]. Wearable technologies may complement these approaches by providing objective data on activity levels and enabling clinicians to monitor changes over time. Such information may support the evaluation of interventions and facilitate more individualized and responsive care.

Participants in this study also emphasized the potential relevance of wearable technologies for monitoring physical activity in outpatient settings. Compared with inpatient environments, outpatient care often involves longer intervals between clinical contacts, which may limit opportunities to monitor changes in patients’ condition. In this context, wearable devices could enable continuous or real-time monitoring of activity levels, helping to bridge gaps between clinical visits and potentially supporting more proactive and personalized care [8]. However, the use of such technologies outside clinical settings may require additional support mechanisms, as patients may face challenges interpreting data or addressing concerns independently. Social implications in outpatient care therefore differ from those in inpatient settings and warrant further in-depth investigation.

By mapping the social implications of wearables to monitor physical activity, this study highlights the importance of considering the broader impact of such devices on patients, healthcare providers, and the care environment [35, 36]. When these implications are carefully addressed during implementation, wearable technologies may offer meaningful opportunities to support the principles and objectives of palliative care.

Understanding these implications requires conceptual frameworks that allow the systematic examination of how health technologies shape different dimensions of care practice. The CARE-HOUSE Framework [35, 36] provided the theoretical background for this study and offered a structured approach to examining the social implications of health technologies in palliative care. The framework´s dimensions informed both the development of the focus group guide and the deductive categories used in the data analysis. This approach supported the identification of social implications, which are often difficult to capture, by delineating relevant areas of care practice in which technologies may generate potential benefits, risks and unintended effects.

Finally, this study was conducted as part of an international collaboration. The diverse expertise of the research team provided perspectives extending beyond a single healthcare system, which supported a broader interpretation of the findings.

### Limitations

Several limitations should be considered when interpreting the findings. First, the study included a relatively small sample recruited from a single research center. Two focus groups were conducted with a total of eight palliative care researchers with clinical experience. Given the exploratory nature of this study, only two focus groups were conducted and data saturation was not claimed [55]. While smaller focus groups can facilitate in-depth discussion [56, 57], the limited number of participants and single-center recruitment restrict the range of perspectives captured and limit the generalizability of the findings.

Second, participants were researchers with clinical experience rather than frontline clinicians actively working in routine care settings. While their combined research and clinical expertise enabled them to reflect on potential social implications from both conceptual and practice-oriented perspectives, their views may not fully represent the experiences of clinicians involved in everyday care delivery. Future research should therefore include frontline healthcare professionals to capture additional practical perspectives on the implementation of wearable technologies in palliative care.

Finally, patients and their family members were not included in this study. As a result, their perspectives on the potential social implications of wearable technologies to monitor physical activity in palliative care are not represented. Future research building on the findings of this study should include patients and caregivers to better understand how such technologies may influence patient experience, autonomy, and relationships within care.

Overall, the findings provide an initial foundation for understanding how wearable technologies may influence care practices in palliative care and highlight considerations for their responsible implementation.

## Conclusion

This paper provides a thoughtful and balanced analysis of the potential social implications of wearables to monitor physical activity in palliative care clinical practice. While these devices offer promising benefits in terms of monitoring and supporting patient activity, their integration into care must be carefully managed to prevent unintended negative consequences. Mapping the social implications of wearables to monitor activity can inform their implementation in palliative care. Addressing these implications is important to ensure that the devices support patient well-being. Future research should focus on translating these insights into practical guidance and incorporate the perspectives of both clinicians and patients.

## Supplementary Information

Supplementary Material 1.

## Data Availability

The datasets used and/or analyzed during the current study are available from the corresponding author on reasonable request.
