# Supplementary material for: Mapping social implications of wearables to monitor physical activity in palliative care – a qualitative approach
Source: BMC Palliat Care. 2026 Jul 2;25:193. doi: 10.1186/s12904-026-02213-x (PMC13330152; doi:10.1186/s12904-026-02213-x)
Supplement: Supplementary file 1 — Supplementary Material 1. [file 12904_2026_2213_MOESM1_ESM.docx]

| **Principles and objectives of palliative care** | Holistic treatment | Support holistic treatment* | | |
| --- | --- | --- | --- | --- |
|  | Prevent and relieve suffering | Prevention* | | |
|  |  | Symptom control* | | |
|  | Improve, maintain quality of life | Being able to do things* | | |
|  |  | Independence/autonomy* | | |
| **Actor level** | Patients | Physical Activity* | Importance of physical activity* | |
|  |  |  | Changes in condition* | |
|  |  |  | Level of activity* | |
|  |  |  | Unrealistic expectations* | |
|  |  |  | Deconditioned* | |
|  |  |  | Performance data is subjective* | |
|  |  |  | Patients do not want to move* | |
|  |  |  | Functional status* | |
|  |  | Life circumstances* | Technology acceptance* | |
|  |  | Burdens and challenges* | Not able to do what family expects* | |
|  |  |  | Fatigued, family pressures* | |
|  |  |  | Contemplating your own mortality* | |
|  |  |  | Anxiety from technology* | |
|  |  |  | Feeling of surveillance* | |
|  |  |  | Watching own decline with technology* | |
|  |  |  | Skin/allergy* | |
|  |  | Needs and preferences* | Technical preferences* | |
|  |  |  | Independence/functional status* | |
|  |  |  | Motivation* | |
|  |  |  | Having people visiting them* | |
|  |  |  | Education & support with technology* | |
|  | Relatives | Needs and preferences* | Want patients to move more/rest more* | |
|  | Health Care professionals | Skills and knowledge* | Skills to advise patients to move* | |
|  |  |  | Experience* | |
|  |  | Burdens and challenges* | Work load* | |
|  |  |  | Missing information on new technology* | |
|  |  | Needs and preferences* | Do not want to use technology* | |
|  |  |  | Getting technology explained* | |
|  |  |  | See benefit of technology/getting engaged* | |
|  | Technology | Design/Location* | Smart watch* | |
|  |  |  | Pedometer (phone)* | |
|  |  |  | Self-report* | |
|  |  |  | Mid-thigh (activPAL)* | |
|  |  |  | Wrist-worn (Actigraphy)* | |
|  |  | Purpose/ What you want out of it* | Give feedback* | |
|  |  |  | Change behaviour* | |
|  |  |  | Retrospective data on physical activity* | |
|  |  | Quality* | Misleading data (activPAL)* | |
|  |  |  | Battery life (activPAL)* | |
|  |  |  | Accuracy (activPAL)* | |
|  |  |  | Missing data (activPAL)* | |
|  |  | Usage* | Usability (Smartwatch)* | |
|  |  |  | Usability (activPAL)* | |
|  |  |  | Tegaderm (activPAL)* | |
|  |  | Functions* | See data from it* | |
|  |  |  | Collected data* | |
|  |  |  | Calculations* | |
|  |  |  | Data output* | |
| **Care level** | Interactions | Patient-physician interaction* | Communication* | |
|  |  |  | Relationship* | |
|  |  |  | Feedback* | |
|  |  |  | Objective movement data* | |
|  |  |  | Data between vistis* | |
|  |  |  | Feeling of surveillance* | |
|  |  |  | Visual impression/ Observation* | |
|  |  | Patient-relatives interaction* | Family conflict about moving* | |
|  |  |  | Spending time with relatives* | |
|  |  | Professionals-relatives-patient interactions* | Professionals as an intermediary between relatives and patient* | |
|  |  |  | Engaging family* | |
|  |  | Human-Technology Interaction* | Being (not) aware of technology* | |
|  |  |  | Affecting behaviour* | |
|  |  |  | Real-time feedback* | |
|  |  |  | Information overload* | |
|  | Roles | Conception of others* | Patients’ self-conception does not align with data* | |
|  |  |  | Relatives’ conception of patient* | |
|  |  |  | Nurses feeling pressured* | |
|  |  | Self-conception* | Professionals* | Competencies* |
|  |  |  |  | Feeling of responsibility* |
|  |  |  | Patients* | Subjective perception of movement* |
|  |  |  |  | Importance of movement for self-conception* |
|  |  |  |  | Self-efficacy, control and motivation* |
|  |  |  |  | Self-conception of being sick* |
|  |  |  |  | Feeling of surveillance* |
|  | Tasks | General tasks in terms of movement* | Encouragement to move* | |
|  |  |  | Self-report* | |
|  |  | Tasks in terms of technology* | Information and explanation* | |
|  |  |  | Application and removement* | Difficulty* |
|  |  |  |  | Responsibility* |
|  |  |  | Skin check* | |
|  |  |  | Data download* | |
|  |  |  | Data usage* | |
|  |  |  | Responsibility* | |
|  |  |  | Additional work* | |
|  |  |  | System management* | |
|  | Processes | Routinely measuring physical activity* | | |
|  |  | Integration in cleaning routine* | | |
| **Context** | Societal | Importance of physical activity* | | |
|  |  | Stigmatizing* | | |
|  |  | Technology acceptance* | | |
|  |  | Culture* | | |
|  |  | Feeling of surveillance* | | |
|  | Structural | Changes in healthcare system* | | |
|  |  | eHealth* | | |
|  |  | Other services* | | |
|  | Organizational | Setting* | | |
|  |  | Skills to advise movement* | | |
|  |  | Implementation in clinical practice* | Training/ Education* | |
|  |  |  | Staff shortage* | |
|  |  |  | Showing the benefit* | |
|  |  |  | Funding* | |
|  |  |  | Data security* | |
|  |  |  | Support* | |
|  |  |  | Infrastructure* | Identification number* |
|  |  |  |  | Software* |
|  | Professional | Physical activity* | | |
|  |  | Complex intervention* | | |
